# Dexmedetomidine inhibits LPS-induced proinflammatory responses via suppressing HIF1α-dependent glycolysis in macrophages

**DOI:** 10.18632/aging.103226

**Published:** 2020-05-20

**Authors:** Qingyuan Meng, Pinhao Guo, Zhengyu Jiang, Lulong Bo, Jinjun Bian

**Affiliations:** 1 Faculty of Anesthesiology, Changhai Hospital, Naval Medical University, Shanghai 200433, China

**Keywords:** dexmedetomidine, glycolysis, HIF1α, macrophage

## Abstract

Dexmedetomidine, a highly selective α2-adrenoceptor agonist, has been reported to exert an anti-inflammatory effect in several animal models, but the mechanism remains unclear. Previous studies have shown that hypoxia inducible factor 1α-induced glycolysis is essential for the activation of inflammatory macrophages. However, whether dexmedetomidine influences hypoxia inducible factor 1α-induced glycolysis and thus exerts an anti-inflammatory effect has been poorly investigated. This study aims to elucidate the anti-inflammatory mechanism of dexmedetomidine involving the hypoxia inducible factor 1α-dependent glycolytic pathway. We showed that dexmedetomidine could suppress lipopolysaccharide-induced inflammatory cytokine production; inhibit the extracellular acidification rate, glucose consumption and lactate production; and decrease the expression of glycolytic genes in macrophages. The enhancement of glycolysis by the granulocyte-macrophage colony-stimulating factor or higher concentration of glucose could reverse the anti-inflammatory effect of dexmedetomidine on lipopolysaccharide-treated macrophages. Moreover, dexmedetomidine significantly inhibited the upregulation of hypoxia inducible factor 1α at the mRNA and protein levels. Genetic inhibition of hypoxia inducible factor 1α expression could reverse the anti-inflammatory effect of dexmedetomidine. Taken together, our results indicate that dexmedetomidine attenuates lipopolysaccharide-induced proinflammatory responses partially by suppressing hypoxia inducible factor 1α-dependent glycolysis in macrophages.

## INTRODUCTION

Macrophages are the frontline cells of innate immunity [1]. They sense and immediately respond to invading pathogens, thus providing an early defense against external attacks. The stimulation of Toll-like receptors (TLRs) on the surface of these cells by microbial products leads to the activation of signaling cascades that result in the induction of antimicrobial genes and inflammatory cytokines [2–5]. These biological factors drive further inflammation and induce the adaptive immune response, which is mediated by effector lymphocytes and is more specific for the particularly invading pathogen [6–8].

Recent studies of cellular metabolism in macrophages have shown profound alterations in metabolic profiles during macrophage activation [9–11]. For example, classically activated macrophages require glycolysis for their survival and polarization [12, 13], whereas oxidative phosphorylation (OXPHOS) favors the differentiation of alternatively activated macrophages [14, 15]. Thus, metabolic reprogramming during macrophage activation is crucial to its function in inflammation and tissue remodeling [16].

Hypoxia inducible factor 1α (HIF1α) is a common component of pathways involved in the control of cellular metabolism and plays an important role in regulating immune cell effector functions [17]. HIF1α facilitates the metabolic switch to glycolysis so that immune cells can continue to produce adenosine triphosphate (ATP) when oxygen is limited, as oxygen is not required for glycolysis. HIF1α promotes this metabolic switch by binding to hypoxia response elements in target genes [18], such as genes encoding the glucose transporter 1 (GLUT1) and glycolytic enzymes [19–21]. HIF1α expression is induced in lipopolysaccharide (LPS)-activated macrophages, where it is critically involved in glycolysis and the induction of proinflammatory gene expression [22].

Dexmedetomidine (DEX), a highly selective agonist of α2-adrenoceptor, is clinically used for sedation and analgesia [23, 24]. Mounting evidence suggests that DEX exhibits anti-inflammatory properties in various sepsis-associated disorders, such as acute lung injury [25], encephalopathy [26], acute kidney injury [27] and microcirculatory dysfunction [28]. However, the mechanism by which DEX exerts an anti-inflammatory effect remains uncertain. Therefore, the aim of the present study was to evaluate the pharmacological effect of DEX on LPS-induced proinflammatory responses in macrophages and explore whether DEX inhibits HIF1α-mediated glycolytic pathway.

## RESULTS

### DEX inhibits the proinflammatory response in LPS-treated macrophages

**Figure 1:**
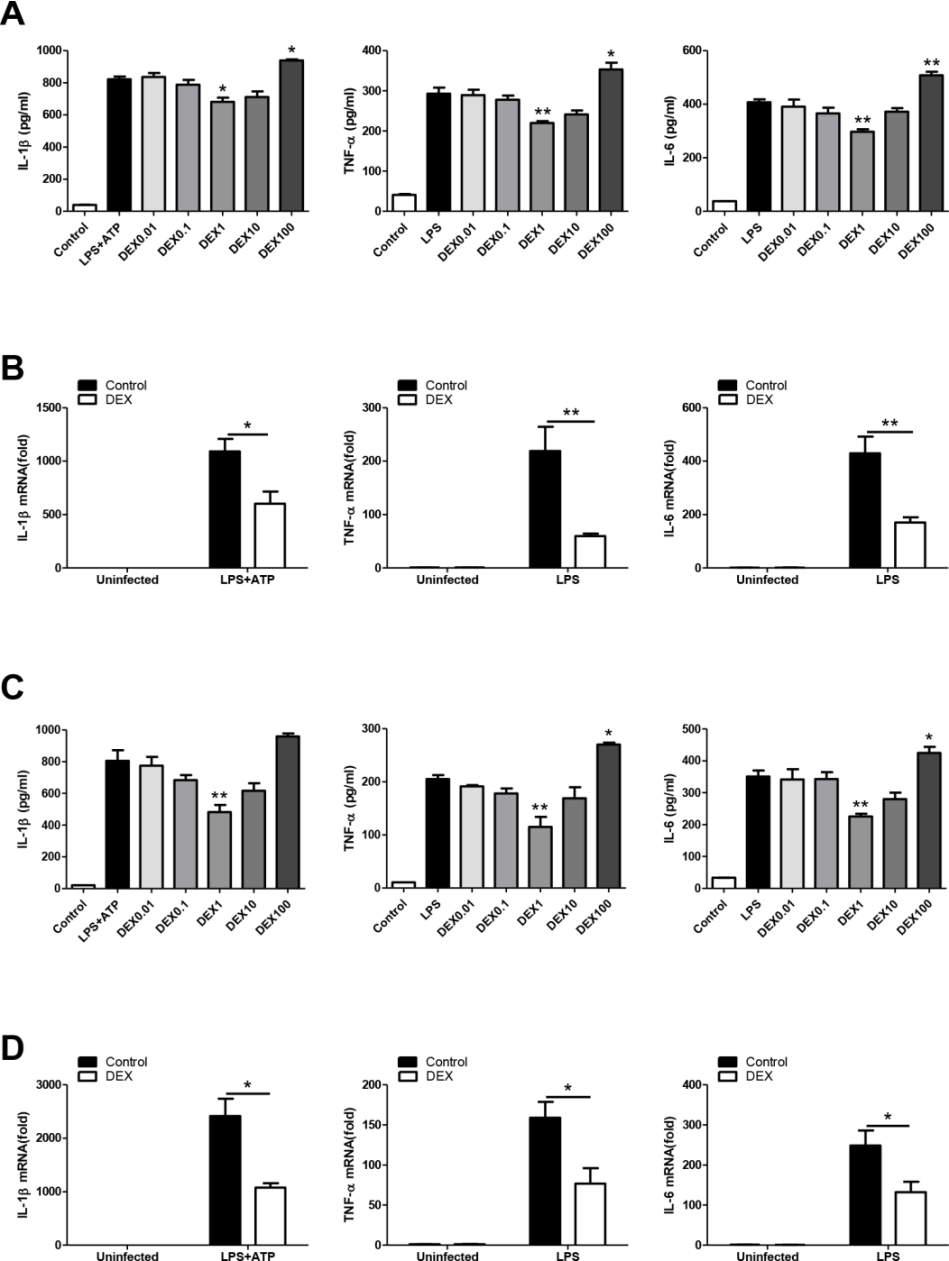
**DEX inhibits the proinflammatory response in LPS-treated macrophages.** (**A**) BMDMs were treated with 100 ng/ml LPS and/or 5 mM ATP and indicated concentrations of DEX for 4h. Levels of IL-1β, TNF-α and IL-6 were determined by ELISA. n = 3; mean ± SEM; * *P* < 0.05; ** *P* < 0.01. (**B**) BMDMs were treated with 100 ng/ml LPS and/or 5 mM ATP and 1 μM DEX for 4 h. The mRNA levels of IL-1β, TNF-α and IL-6 were determined by real-time PCR. n = 3; mean ± SEM; * *P* < 0.05; ** *P* < 0.01. (**C**) PMs were treated with 100 ng/ml LPS and/or 5 mM ATP and indicated concentrations of DEX for 4 h. Levels of IL-1β, TNF-α and IL-6 were determined by ELISA. n = 3; mean ± SEM; ** *P* < 0.01. (**D**) PMs were treated with 100 ng/ml LPS and/or 5 mM ATP and 1 μM DEX for 4 h. The mRNA levels of IL-1β, TNF-α and IL-6 were determined by real-time PCR. n = 3; mean ± SEM; * *P* < 0.05.

In this study, different types of macrophages were used to test whether DEX has anti-inflammatory effect as previously reported [29, 30]. We found that DEX at 1μM significantly suppressed the LPS-induced upregulation of IL-1β, TNFα and IL-6 after LPS administration in BMDMs, whereas DEX at 100μM promoted the production of these cytokines (Figure 1A). We measured cell viability after treatment with graded DEX using the CCK-8 assay and found that DEX at 100μM had a significant inhibitory effect on cell viability (Supplementary Figure 1). We also showed that DEX at 1μM significantly suppressed LPS-induced mRNA upregulation of the cytokines in BMDMs (Figure 1B). The decreased expression of IL-1β, TNFα and IL-6 with 1μM DEX treatment was also observed in LPS-treated PMs (Figure 1C, 1D). Consistent with findings of previous studies, our results demonstrate that DEX suppresses proinflammatory responses in LPS-primed macrophages.

### DEX inhibits glycolysis in LPS-treated macrophages

**Figure 2:**
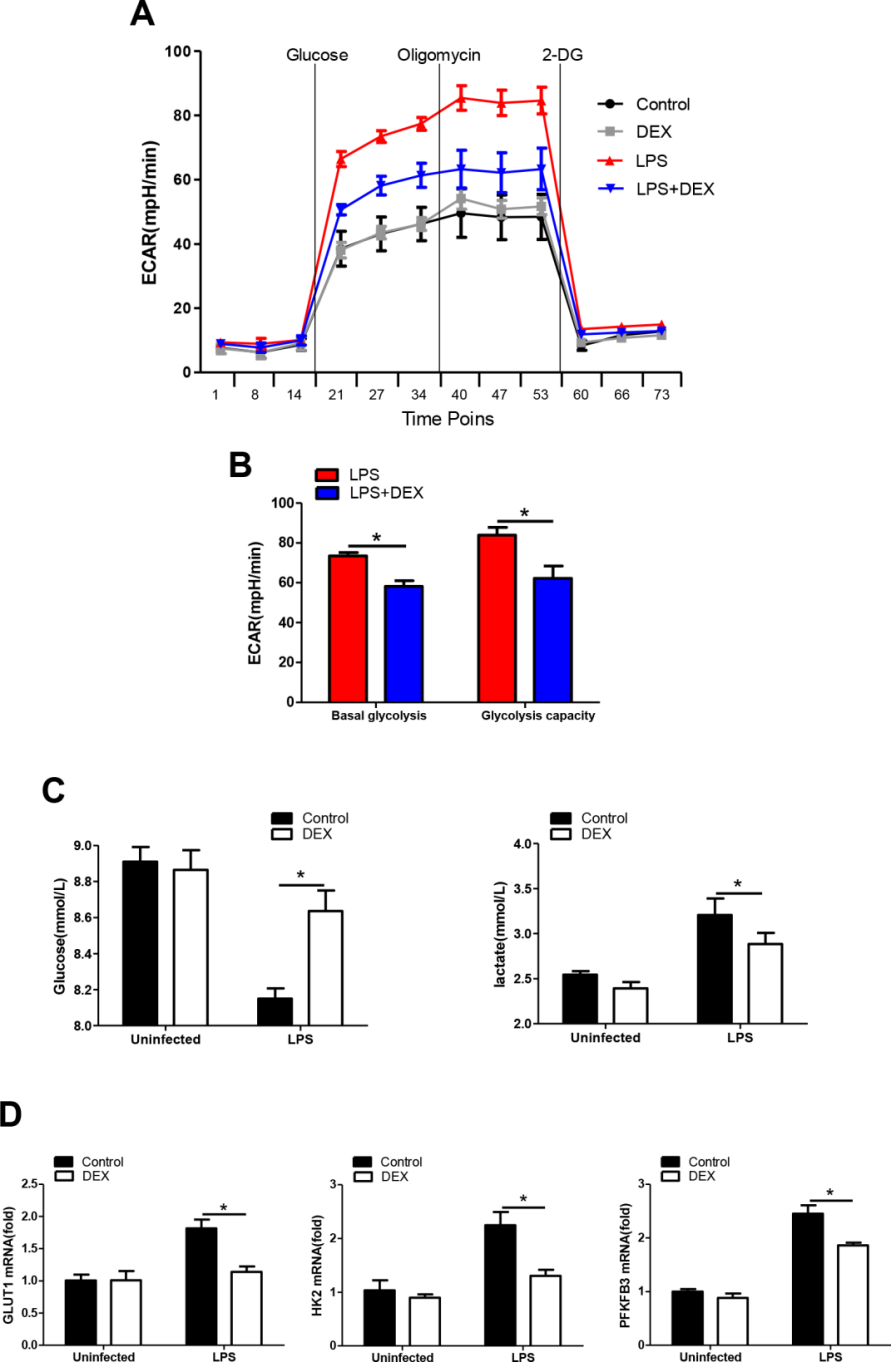
**DEX inhibits glycolysis in LPS-treated macrophages.** (**A** and **B**) BMDMs were seeded in Seahorse XFe96 cell culture microplates and treated with 100 ng/ml LPS and 1 μM DEX for 4 h. The real-time ECAR was recorded, and basal glycolysis and glycolytic capacity values were plotted. n = 5; mean ± SEM; * *P* < 0.05. (**C**) BMDMs were treated with 100 ng/ml LPS and 1 μM DEX for 4 h. Supernatants were collected, and the levels of glucose and lactate were measured. n = 3; mean ±SEM; * *P* < 0.05. (**D**) BMDMs were treated with 100 ng/ml LPS and 1 μM DEX for 4 h. The mRNA levels of GLUT1, HK2 and PFKFB3 were determined by RT-PCR. n = 3; mean ± SEM; * *P* < 0.05.

It has been increasingly recognized that augmented aerobic glycolysis is essential to the development of a proinflammatory phenotype in LPS-primed macrophages [31]. We hypothesize that DEX inhibits inflammation by suppressing glycolysis in macrophages. Thus, BMDMs were analyzed to determine changes in the ECAR, a measure of glycolysis, after LPS stimulation with or without DEX. We found that the ECAR in LPS-treated BMDMs was markedly increased compared with that in control cells, while DEX suppressed this elevation (Figure 2A, 2B). These data were in line with the lower levels of glucose consumption and lactate production found in BMDMs treated with LPS and DEX, compared with those treated with LPS alone (Figure 2C). GLUT1 plays an important role in glucose uptake in macrophages during LPS stimulation [32]. Hexokinase-II (HK2) and 6-Phosphofructo-2-kinase/fructose-2,6-bisphosphatase (PFKFB3) are rate-limiting enzymes of glycolysis and are indispensable for the induction of glycolysis in activated innate immune cells [33, 34]. Indeed, our data showed that BMDMs predominantly expressed GLUT1, HK2 and PFKFB3 at the inflammatory state, whereas this upregulation was alleviated after DEX treatment (Figure 2D). Collectively, our findings suggest that DEX inhibits glycolysis in macrophages by suppressing glycolytic flux.

### Enhancement of glycolysis reverses the anti-inflammatory effect of DEX in LPS-treated macrophages

**Figure 3:**
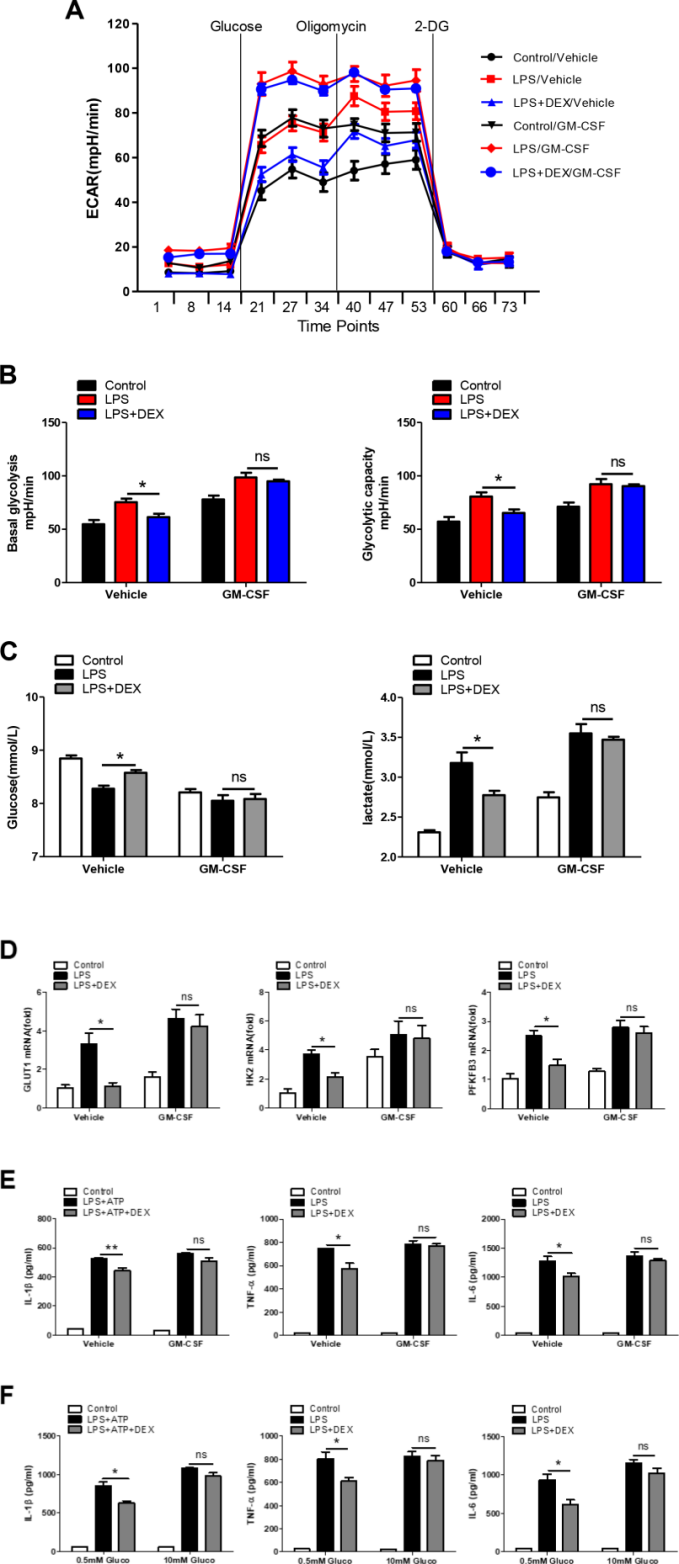
**Enhancement of glycolysis reverses the anti-inflammatory effect of DEX on LPS-treated macrophages.** (**A** and **B**) BMDMs were seeded in Seahorse XFe96 cell culture microplates and treated with 25 ng/ml GM-CSF for 24 h before being treated with 100 ng/ml LPS and 1 μM DEX for 4 h. The real-time ECAR was recorded, and basal glycolysis and glycolytic capacity values were plotted. n = 5; mean ± SEM; * *P* < 0.05. (**C**) BMDMs were treated with 25 ng/ml GM-CSF for 24 h before being treated with 100 ng/ml LPS and 1 μM DEX for 4 h. Supernatants were collected, and the levels of glucose and lactate were measured. n = 3; mean ±SEM; * *P* < 0.05. (**D**) BMDMs were treated with 25 ng/ml GM-CSF for 24 h before being treated with 100 ng/ml LPS and 1 μM DEX for 4 h. The mRNA levels of GLUT1, HK2 and PFKFB3 were determined by RT-PCR. n = 3; mean ± SEM; * *P* < 0.05. (**E**) BMDMs were treated with 25 ng/ml GM-CSF for 24 h before being treated with 100 ng/ml LPS and/or 5 mM ATP and 1 μM DEX for 4 h. Levels of IL-1β, TNF-α and IL-6 were measured by ELISA. n = 3; mean ± SEM; * *P* < 0.05; ** *P* < 0.01. (**F**) BMDMs were treated with 0.5mM or 10mM glucose before being treated with 100 ng/ml LPS and/or 5 mM ATP and 1 μM DEX for 4 h. Levels of IL-1β, TNF-α and IL-6 were measured by ELISA. n = 3; mean ± SEM; * *P* < 0.05.

To further determine if the inhibition of glycolysis by DEX accounted for the weaker LPS-induced inflammatory responses in macrophages, GM-CSF was added to evaluate the cellular glycolysis. Our data showed that GM-CSF almost completely blunted the DEX-induced decrease in the ECAR (Figure 3A and 3B), reductions in glucose consumption and lactate production (Figure 3C), and downregulation of glycolysis-related gene expression (Figure 3D), suggesting that the inhibition of glycolysis by DEX was abolished by GM-CSF pretreatment. We also found that the reduction in the expression of IL-1β, TNFα and IL-6 in DEX-treated macrophages was reversed (Figure 3E). Moreover, we measured the production of IL-1β, TNFα and IL-6 by adding different concentrations of glucose. Results showed that the inhibitory effect of DEX was alleviated in the presence of a higher and saturating concentration of glucose (10mM) (Figure 3F). Taken together, these results indicate that enhancing glycolysis could reverse the anti-inflammatory effect of DEX on LPS-treated macrophages.

### HIF1α is required to regulate the anti-inflammatory effect of DEX in LPS-treated macrophages

**Figure 4:**
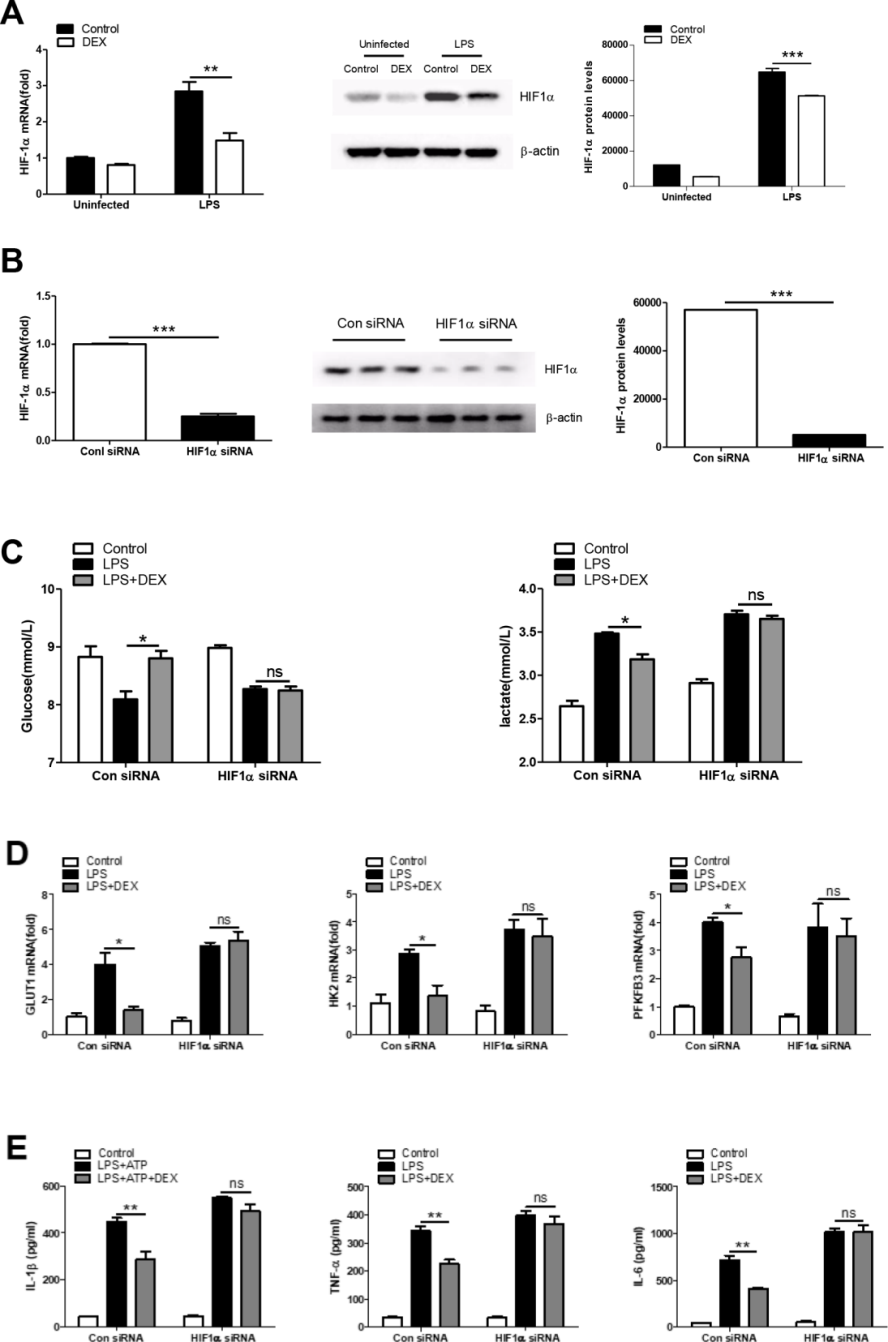
**HIF1α is required for regulating the anti-inflammatory effect of DEX on LPS-treated macrophages.** (**A**) BMDMs were treated with 100 ng/ml LPS and 1 μM DEX for 4 h. The mRNA and protein levels of HIF1α were determined by RT-PCR and Western blotting, respectively. n = 3; mean ± SEM; ** *P* < 0.01, *** *P* < 0.001. (**B**) BMDMs were transfected with HIF1α siRNA or Negative control siRNA for twenty-four hours. The mRNA and protein levels of HIF1α were determined by real-time PCR and Western blotting, respectively. n = 3; mean ± SEM; *** *P* < 0.001. (**C**) BMDMs were transfected as in (**B**). Twenty-four hours after transfection, the cells were treated with 100 ng/ml LPS and 1 μM DEX for 4 h. Supernatants were collected, and the levels of glucose and lactate were measured. n = 3; mean ± SEM; * *P* < 0.05. (**D**) BMDMs were transfected as in (**B**). Twenty-four hours after transfection, the cells were treated with 100 ng/ml LPS and 1 μM DEX for 4 h. The mRNA levels of GLUT1, HK2 and PFKFB3 were determined by RT-PCR. n = 3; mean ± SEM; * *P* < 0.05. (**E**) BMDMs were transfected as in (**B**). Twenty-four hours after transfection, the cells were treated with 100 ng/ml LPS and/or 5 mM ATP and 1 μM DEX for 4 h. Levels of IL-1β, TNF-α and IL-6 were determined by ELISA. n = 3; mean ± SD; ** *P* < 0.01.

Given that HIF1α is a key metabolic regulator which plays an important role in inflammation [35], we asked whether the regulation of cellular metabolism by HIF1α is essential for the anti-inflammatory effects of DEX. First, we found that DEX inhibited the LPS-induced expression of HIF1α at both the mRNA and protein levels in BMDMs (Figure 4A). We next tested whether the inhibition of HIF1α by DEX was responsible for the effects of DEX on glycolysis and inflammation in LPS-induced macrophages. HIF1α was knocked down in BMDMs by special small-interfering RNA (siRNA) and the success of the knockdown was confirmed (Figure 4B). We found that HIF1α knockdown reversed the inhibitory effects of DEX on LPS-induced increases in glucose consumption, lactate production (Figure 4C) and glycolysis-associated gene expression (Figure 4D). In addition, HIF1α knockdown reversed the inhibition of IL-1β, TNFα and IL-6 expression by DEX in LPS-treated macrophages (Figure 4E). Collectively, these data suggest that DEX inhibits the production of inflammatory cytokines partially by suppressing HIF1α activation in macrophages.

## DISCUSSION

Macrophages respond to microbial stimuli by triggering the expression of an array of inflammatory cytokines, which in turn cause the infiltration and activation of other types of immune cells to orchestrate a full-fledged immune-inflammatory response [36, 37]. Proinflammatory stimuli induces a metabolic switch in macrophages, leading to a Warburg-like upregulation of aerobic glycolysis to regulate the balance between inflammatory and regulatory immune phenotypes [12, 38]. Here, we provide evidence for the DEX-mediated regulation of glucose metabolism in activated macrophages and suggest that DEX acts to inhibit inflammatory responses in part by controlling the HIF1α-dependent glycolytic pathway.

DEX has been regarded as a highly selective α2-adrenoceptor agonist and is mostly applied in different clinical settings for sedative or analgesic requirements. Along with its beneficial effects, DEX has been reported to potentially exert anti-inflammatory effects during endotoxemia. A previous study revealed that DEX significantly reduces mortality and decreases the levels of inflammatory cytokines during endotoxemia in rats [39]. DEX reduces sepsis-related acute lung injury and has a protective effect on ischemia-reperfusion injury of the heart, brain, kidneys, and intestine in animal model [40–43]. DEX affects the immune cell ratio and suppresses inflammatory cytokine production in spleen and lymphocytes [44]. Our results were consistent with findings of previous studies, showing that DEX at 1μM significantly reduces the production and release of proinflammatory cytokines by LPS-induced macrophages. The dose of DEX used in our study is much higher than its clinical use. Because of interspecies variability, drug doses needed in animal studies are usually much higher than that used clinically (often up to 10-fold higher). Our data showed that the dose of DEX used did not cause any damage to cell viability. However, the safety of using such a high dosage of DEX in septic patients remains to be elucidated. Nevertheless, it was reported that some patients, especially pediatric patients, required higher dosages of DEX (up to approximately 5 to 10 times of clinical dosages in adults) to achieve adequate sedation. Moreover, such dosages were well-tolerated by those patients [29, 30].

The activation of macrophages by proinflammatory stimuli causes them to undergo a metabolic switch towards glycolysis and away from OXPHOS. The importance of glycolysis in the proinflammatory response of macrophages has been demonstrated in a previous study, whereas inhibition of glycolysis using 2-deoxyglucose (2-DG) decreased the proinflammatory response [13]. Gong et al. indicated that blockade of glycolysis with 3-(3-pyridinyl)-1-(4-pyridinyl)-2-propen-1-one (3PO) could alleviate sepsis-related acute lung injury via suppressing inflammation and apoptosis of alveolar epithelial cells [45]. Xie et al. indicated that inhibition of pyruvate kinase 2 (PKM2), which catalyzes the final and rate-limiting reaction of the glycolytic pathway, could attenuate the NOD-, leucine rich region- and pyrin domain-containing-3 (NLRP3) and absent in melanoma 2 (AIM2) inflammasome activation and consequently suppress the release of IL-1β and high mobility group box 1 (HMGB1) [46]. Based on studies mentioned above, we speculate that DEX might regulate glycolysis in activated macrophages. Our data showing the restraint of glycolysis by DEX via the suppression of glucose uptake, lactate production and glycolytic gene expression suggest that DEX might act to reverse the metabolic program associated with the inflammatory response.

We next asked whether the inhibition of aerobic glycolysis mediated the immunological actions of DEX. GM-CSF has been reported to augment glycolytic flux via a mechanism that depends on PFKFB3 in vitro [47]. Na et al. showed that GM-CSF increases macrophage glycolytic capacity by upregulating GLUT expression [48]. In this study, we showed that GM-CSF pretreatment almost completely reversed the attenuating effects of DEX on the LPS-induced enhancement of glycolysis and release of inflammatory cytokines. It should be noted that this result could not be completely attributed to the acceleration of glycolysis, for that GM-CSF might have other effects through which it can promote LPS-induced inflammatory cytokine expression. Therefore, we next measured the production of IL-1β, TNFα and IL-6 in the presence of a saturating concentration of glucose and found that DEX was much less effective under this condition, suggesting that the anti-inflammatory effect of DEX can be attenuated by enhancing glycolysis.

**Figure 5:**
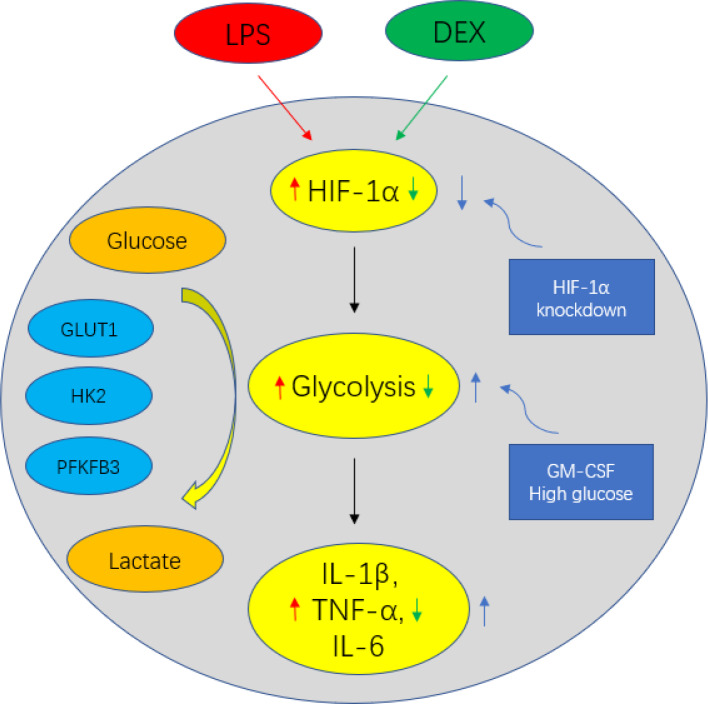
**Schematic figure representing DEX-mediated anti-inflammatory response in LPS-treated macrophages.** DEX inhibits the production of IL-1β, TNF-α and IL-6 via suppressing HIF1α expression and the upregulation of glycolysis in LPS-treated macrophages. However, enhancing glycolysis by GM-CSF could reverse the anti-inflammatory effect of DEX on LPS-treated macrophages. Moreover, the restraint of IL-1β, TNF-α and IL-6 production by DEX was abolished by decreasing HIF1α levels genetically with HIF1α knockdown.

It is increasingly recognized that HIF1α acts as a central regulator of cellular metabolism and promotes inflammatory gene expression. Blouin et al. were the first to show that the stimulation of macrophages with LPS increases HIF1α protein levels, leading to the formation of a functional HIF-1 complex that binds to hypoxic response elements in target genes, including GLUT1, HK2 and PFKFB3 [49]. It was later found that HIF1α-mediated glycolytic reprogramming of activated macrophages plays a significant role in monocyte-derived macrophage migration into tissues [50]. HIF1α also induces the transcription of the inflammatory cytokines. Our results indicated that DEX inhibited HIF1α expression, which implied that DEX controlled metabolic processes by engaging in the regulation of HIF1α signaling. Our conjecture described above was supported by the finding that the DEX-mediated inhibition of glycolysis was reversed after HIF1α knockdown. In addition, we showed that the restraint of IL-1β, TNFα and IL-6 production by DEX was abolished by decreasing HIF1α levels genetically with HIF1α knockdown. DEX may inhibit cerebral ischemia-reperfusion (I/R) injury by inhibiting the HIF1α pathway and inhibiting apoptosis in I/R rat brain. The inhibition of HIF1α by DEX restored the balance between catabolic aerobic processes and catabolic anaerobic processes [51]. Our results indicate that DEX inhibits glycolysis by suppressing HIF1α, which was consistent with the previous study [51]. Collectively, these data suggest that HIF1α is necessary for DEX to exert its anti-inflammatory effect. Figure 5 depicts the overall mechanism of glucose metabolic regulation of DEX in LPS-treated macrophages.

However, there are several limitations to be noted here. First, although we found that DEX regulates the expression of HIF1α, the precise mechanism remains elusive. A key pathway for HIF1α activation by LPS involves mammalian target of rapamycin (mTOR). Hence, we speculate that DEX suppresses HIF1α expression in part by inhibiting mTOR activation and deserve further investigation. Second, there is a lack of evidence in vivo to support the inhibition of glycolysis by DEX. Thus, in vivo studies with animal models were warranted in future.

To conclude, our study reveals a new mechanism by which DEX regulates cellular metabolism through HIF1α inhibition. We propose that this metabolic regulation by DEX is critical to inflammation control and deserves further investigation.

## MATERIALS AND METHODS

### Reagents

LPS from *Escherichia coli* O111:B4, ATP (A2383) and DEX (SML0956) were obtained from Sigma-Aldrich. GM-CSF and M-CSF were purchased from PeproTech.

### Cell culture and animals

Bone marrow-derived cells from C57BL/6J mice were cultured in RPMI 1640 medium (Invitrogen) supplemented with 10% FBS and 1% penicillin/streptomycin and differentiated into bone marrow-derived macrophages (BMDMs) with murine macrophage colony-stimulating factor (M-CSF) treatment for 5 d. Peritoneal macrophages (PMs) were elicited in C57BL/6J mice 3 d after the intraperitoneal injection of 4% thioglycolate (Sigma-Aldrich) and cultured in RPMI 1640 medium without glucose. Male C57BL/6 mice were obtained from the Laboratory Animal Center of Naval Medical University and housed in a specific pathogen-free environment at the optimal temperature with a 12 h light/dark cycle.

### Small-interfering RNA (siRNA) treatment

The siRNA sequences were designed at GeneChem (Shanghai, China). The following siRNA sequences were used: HIF1α siRNA: 5’-GCUCACCAUCAGUUAUUUATT-3’, and Negative control siRNA: 5’-UUCUCCGAACGUGUCACGUTT-3’. Murine BMDMs were transfected with the siRNAs using Lipofectamine 2000 (Invitrogen) according to the manufacturer’ instructions. The supernatant was replaced with complete culture medium after 24 h.

### Extracellular acidification rate (ECAR)

The XFe96 Extracellular Flux Analyzer (Agilent) was used for real-time recording of the ECAR. In brief, BMDMs were seeded in Seahorse XFe96 microplates (3×10^4^ cells per well) and treated with LPS, DEX, or both. Before analysis, the cells were incubated in ECAR medium for 1 h at 37°C in room air. The cells were sequentially treated with 10 mM glucose, 1 μM oligomycin, and 50 mM 2-DG. Real-time ECARs were recorded according to the manufacturer’s manual.

### Metabolite measurements

The glucose and lactate concentrations within cell medium were determined by the Glucose and Lactate Colorimetric Assay Kits (BioVision), respectively.

### Cell Counting Kit-8 assay

The viability of BMDMs were determined using the Cell Counting Kit-8 assay kit (Dojindo) as previously reported [52].

### Real-time polymerase chain reaction (PCR)

Total RNA was extracted from cells using RNAiso (TaKaRa) according to the manufacturer’s instructions. cDNA was synthesized using a PrimeScript^TM^RT reagent kit (TaKaRa). Real-time PCR was performed using SYBR green master mix (TaKaRa). The real-time PCR primers used in this study are listed in Supplementary Table 1 of the Supplementary Materials.

### Enzyme-linked immunosorbent assay (ELISA)

Levels of IL-1β, TNFα and IL-6 in cell supernatants were measured with commercial ELISA kits (eBioscience) according to the manufacturer’s instructions.

### Western blotting

Cells were homogenized in lysis buffer, and protein lysates were separated on 10% SDS gels and transferred to polyvinylidene fluoride membranes (Millipore). After blocking, the membranes were incubated with a primary antibody (anti-HIF1α, Cell Signaling Technology) overnight, followed by a 1 h incubation with a horseradish peroxidase-conjugated secondary antibody (Cell Signaling Technology) at room temperature. The band intensity was quantified by densitometric analyses using ImageJ software.

### Statistical analysis

Data are presented as the mean ± standard deviation. Significant differences between multiple groups were detected using ANOVA. Differences between two groups were detected using a *t* test. All analyses were performed using GraphPad Prism 5 statistical software. A value of *P* < 0.05 was considered statistically significant.

## Supplementary Material

Supplementary Figure 1

Supplementary Table 1
