# Supplementary material for: Dexmedetomidine inhibits LPS-induced proinflammatory responses via suppressing HIF1α-dependent glycolysis in macrophages
Source: Aging (Albany NY). 2020 May 20;12(10):9534–48. doi: 10.18632/aging.103226 (PMC7288940; doi:10.18632/aging.103226)
Supplement: Supplementary Figure 1 [file aging-12-103226-s001..pdf]

SUPPLEMENTARY FIGURE

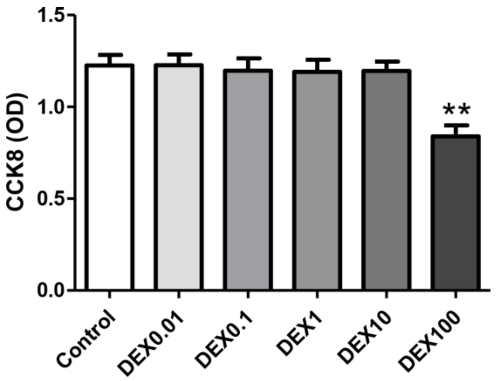

Supplementary Figure 1. Effect of DEX at different concentrations on BMDMs viability after incubation for 24h. BMDMs were treated with graded concentrations of DEX (0.01, 0.1, 1, 10, and 100μM) for 24h. The cell viability of BMDMs were determined using the CCK-8 assay. n = 3; mean ± SEM; ** *P* < 0.01.
