# Supplementary material for: Dexmedetomidine inhibits LPS-induced proinflammatory responses via suppressing HIF1α-dependent glycolysis in macrophages
Source: Aging (Albany NY). 2020 May 20;12(10):9534–48. doi: 10.18632/aging.103226 (PMC7288940; doi:10.18632/aging.103226)
Supplement: Supplementary Table 1 [file aging-12-103226-s002..pdf]

SUPPLEMENTARY TABLE

Supplementary Table 1. Primers for RT-PCR.

Genes	Forward primers (5'-3')	Reverse primers (5'-3')
TNFα	aatggcctccctctcatcag	cccttgaagagaacctggga
IL-6	taccactcccaacagacctg	ggtactccagaagaccagagg
GLUT1	cagttcggctataacactggtg	gcccccgacagagaagatg
HK2	tgatcgcctgcttattcacgg	aaccgcctagaaatctccaga
PFKFB3	agaagctgactcgctacctc	aaggcacactgttttcggac
HIF1α	tcaagtcagcaacgtggaag	tatcgaggctgtgtcgactg
B2M	cggcctgtatgctatccaga	gggtgaattcagtgtgagcc
